# Mechanism of Zuogui pill in the treatment of polycystic ovary syndrome based on LC-MS and proteomics

**DOI:** 10.1186/s13048-025-01802-3

**Published:** 2025-09-26

**Authors:** Yuxuan Ke, Yiting Tang, Jiajing He, Min Xiao, Min Zhao

**Affiliations:** 1 School of Acupuncture-Moxibustion and Orthopedics, Hubei University of Chinese Medicine, Wuhan, 430065 China; 2 School of Basic Medical Sciences, Hubei University of Chinese Medicine, Wuhan, 430065 China; 3 School of Chinese Medicine, Hubei University of Chinese Medicine, Wuhan, 430065 China; 4 Hubei Shizhen Laboratory, Wuhan, 430061 China

**Keywords:** ZuoGui pill, Polycystic ovary syndrome, 4D-DIA-based proteomics, MAPK signaling pathway

## Abstract

**Background:**

Polycystic ovary syndrome (PCOS) is a common endocrine and metabolic disorder that severely impairs female fertility. Zuogui pill (ZGP) is clinically used for the treatment of reproductive disorders such as menopause syndrome and premature ovarian failure, improving female reproductive health. However, the mechanism of its action in treating PCOS remains unclear. This study aimed to investigate the mechanism of ZGP in treating PCOS based on liquid chromatography-mass spectrometry (LC-MS) and proteomics.

**Methods:**

LC-MS was utilized to analyze and identify the active components of ZGP. A PCOS rat model was established using a high-fat diet combined with letrozole to assess the efficacy of ZGP. Serum hormone levels in rats were detected using ELISA, and ovarian morphology was examined via hematoxylin and eosin staining. 4D-DIA-based proteomics was performed on ovarian samples from rats, and a combined analysis with network pharmacology was conducted to identify key pathways and their targets. Finally, Western blot analysis was used to validate the analytical results.

**Results:**

A total of 31 components of ZGP were identified through LC-MS analysis combined with the TCMSP database. In the PCOS rat model, ZGP significantly improved obesity and ovarian tissue damage, regulated serum sex hormone secretion levels. Through a combined analysis of network pharmacology and 4D-DIA-based proteomics, the potential mechanism of ZGP in improving PCOS was determined to be the mitogen-activated protein kinase (MAPK) signaling pathway. WB analysis revealed that ZGP reversed the expression levels of p-ERK1/2 and p-JNK.

**Conclusion:**

This study demonstrates that ZGP may exert a therapeutic effect on PCOS through the MAPK signaling pathway.

**Supplementary Information:**

The online version contains supplementary material available at 10.1186/s13048-025-01802-3.

## Introduction

Polycystic ovary syndrome (PCOS) is one of the most common endocrine and metabolic disorders among women of reproductive age, affecting 8–13% of women globally, according to statistics. Its clinical manifestations primarily include hyperandrogenism, luteinizing hormone (LH) hypersecretion, infertility, polycystic ovaries, and insulin resistance (IR) [1], significantly impacting women’s physical and mental health and quality of life. Metformin, as the most commonly used insulin sensitizer, has been employed in the treatment of PCOS patients with endocrine disturbances. While it can improve menstrual cycle irregularities and reduce subcutaneous fat, its common side effects, such as diarrhea and abdominal discomfort, limit its clinical application [2]. Clinical studies have found that the use of traditional Chinese medicine (TCM) prescriptions or integrated traditional Chinese and Western medicine for the treatment of PCOS can often reduce the incidence of the aforementioned adverse reactions, significantly enhancing patients’ quality of life [3].

In recent years, the application of TCM in the treatment of PCOS has gradually gained attention and recognition [4]. TCM, with minimal adverse reactions, is widely used in the treatment of gynecological diseases [5]. Unlike Western medicine, in TCM theory, the “kidney” is not only related to the anatomical kidney but is also closely linked to women’s reproductive health. The kidney governs reproduction, and kidney deficiency often leads to disorders in women’s reproductive function, manifesting as irregular menstruation, amenorrhea, infertility, and other symptoms [6]. Although PCOS does not have a direct corresponding term in TCM theory, its symptoms and manifestations align with those of diseases caused by kidney deficiency described in TCM. The TCM theory suggests that kidney deficiency is one of the main pathogeneses of PCOS [7]. The “Kidney-Tiangui-Chongren” axis in TCM is analogous to the hypothalamic-pituitary-ovarian (HPO) axis in modern medicine [8]. Dysfunction of the HPO axis is one of the pathogeneses of PCOS, leading to increased follicle-stimulating hormone (FSH) and LH, which in turn affect ovarian androgen synthesis and folliculogenesis [9].

Clinical treatment has found that TCM prescriptions can improve PCOS-related symptoms, including endocrine and metabolic abnormalities, infertility, obesity, and hirsutism [10]. Zuogui pill (ZGP), derived from *Jingyue Quanshu (Complete Compendium of Zhang Jingyue)* and created by the physician Zhang Jingyue in Ming dynasty, possesses the efficacy of supplementing essence and marrow. It is currently widely used in the treatment of various diseases caused by kidney deficiency [11]. Studies have shown that ZGP can improve ovarian weight, increase serum anti-Müllerian hormone (AMH) and estradiol (E2) levels, reduce inflammation, and promote the proliferation and differentiation of ovarian stem cells caused by chemotherapy [12, 13]. Additionally, ZGP can promote the recovery of ovarian function by increasing the expression of GDF-9 and SMAD2 proteins [14].

**Fig. 1:**
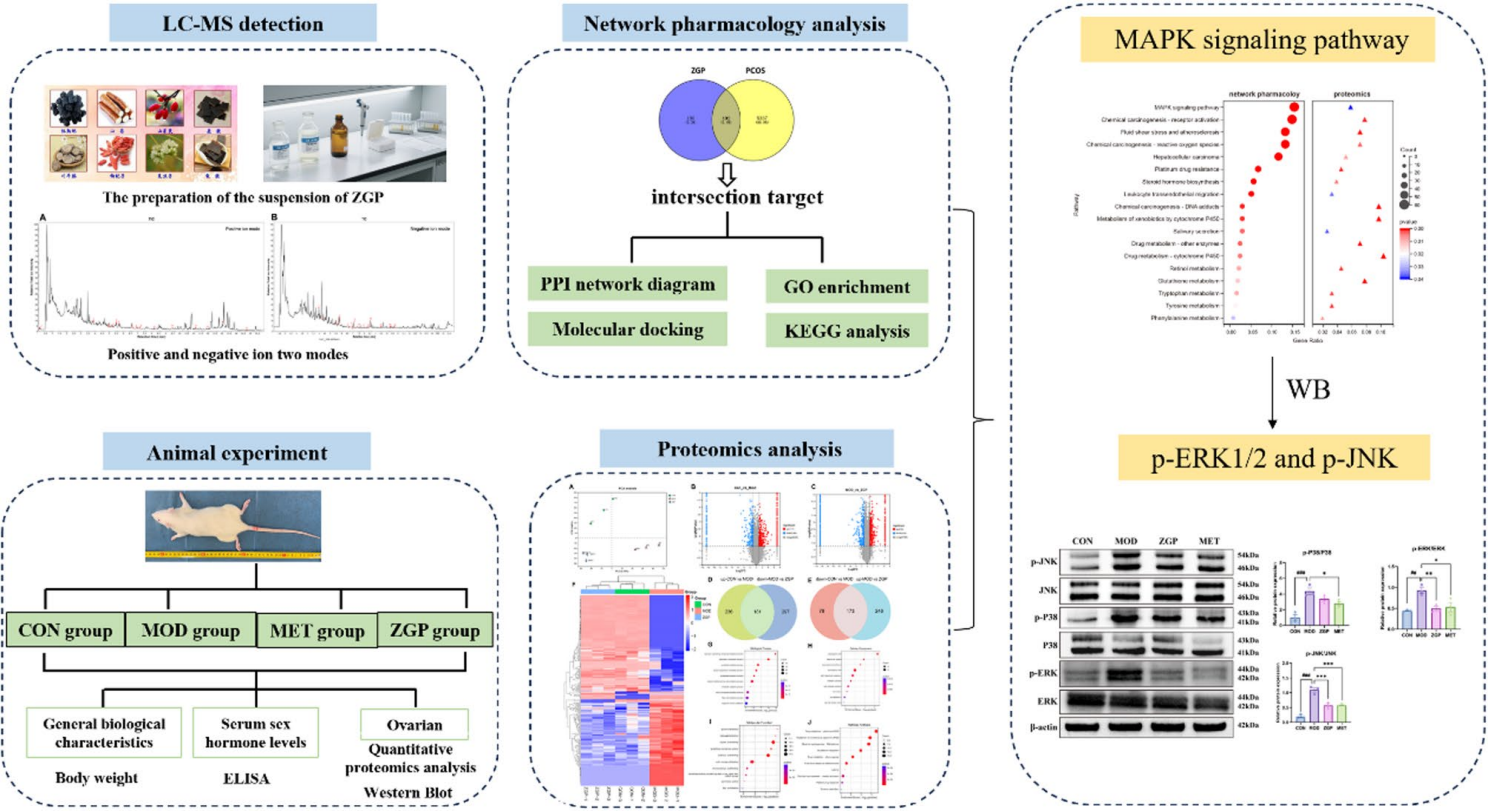
The entire workflow of the study

Based on the above findings, this study integrates network pharmacology, 4D-DIA-based proteomics, animal experiments, and other techniques to deeply investigate the therapeutic effects of ZGP on PCOS (Fig. 1). Through multi-angle experimental data, this study revealed the mechanism of action of ZGP in the treatment of PCOS and provided strong data support for its clinical application.

## Materials and methods

### Experimental materials

All reagents used in the experiment were of chromatographic or analytical grade. The main experimental materials and reagents are listed in Table S1.

### Drug preparation

Letrozole was purchased from Jiangsu Hengrui Pharmaceuticals Co., Ltd., with drug approval number: H19991001.

Metformin aqueous solution: Metformin was obtained from Merck & Co., Inc., with drug approval number: H20023370. Metformin tablets were ground into powder using a mortar and dissolved in double-distilled water to prepare an aqueous solution with a drug dose of 300 mg·kg⁻¹.

ZGP suspension: ZGP was purchased from Zhongjing Wanxi Pharmaceutical Co., Ltd., with drug approval number: Z41020696. Based on the body surface area conversion formula between rats and humans, the calculated dosage of ZGP was 1.62 g·kg⁻¹. The ZGP was ground into powder using a mortar and dissolved in double-distilled water to prepare a solution of the required concentration.

### LC-MS

#### Sample processing

ZGP suspension were first mixed with grinding beads and an internal standard extraction solution (methanol: water = 4:1). Subsequently, they were grinded at a temperature of -10 °C and a frequency of 50 Hz for a duration of 6 min. Following this, the samples underwent ultrasonic extraction for 30 min and were then allowed to settle statically at -20 °C for another 30 min. After this settlement period, the samples were centrifuged at 4 °C for 15 min, and the resulting supernatant was collected for further analysis. 20 ul supernatant from each sample were mixed to prepare quality control (QC) samples.

#### LC-MS detection

The LC-MS/MS analysis of sample was conducted on a Thermo UHPLC-Q Exactive HF-X system equipped with an ACQUITY HSS T3 column (100 mm × 2.1 mm i.d., 1.8 μm; Waters, USA) at Majorbio Bio-Pharm Technology Co. Ltd. (Shanghai, China). The mobile phases consisted of 0.1% formic acid in water: acetonitrile (95:5, v/v) (solvent A) and 0.1% formic acid in acetonitrile: isopropanol: water (47.5:47.5, v/v) (solvent B). The flow rate was 0.40 mL/min and the column temperature was 40℃ [15].

The mass spectrometric data were collected using a Thermo UHPLC-Q Exactive HF-X Mass Spectrometer equipped with an electrospray ionization (ESI) source operating in positive mode and negative mode. The optimal conditions were set as followed: source temperature at 425℃; sheath gas flow rate at 50 arb; Aux gas flow rate at 13 arb; ion-spray voltage floating (ISVF) at-3500 V in negative mode and 3500 V in positive mode, respectively; Normalized collision energy, 20-40-60 V rolling for MS/MS. Full MS resolution was 60,000, and MS/MS resolution was 7500. Data acquisition was performed with the Data Dependent Acquisition (DDA) mode. The detection was carried out over a mass range of 70–1050 m/z.

Raw data were imported into the metabolomics processing software (Progenesis QI v3.0) for baseline filtering, peak identification, integral, retention time correction, peak alignment, etc., ultimately yielding a data matrix containing information such as retention time, mass-to-charge ratio, and peak intensity. Simultaneously, a library search for characteristic peaks was conducted by matching the acquired mass spectrometry (MS) data against a proprietary metabolite database for traditional Chinese medicines (MJBIOTCM) established on the Majorbio. The mass error tolerance was set to less than 10 ppm, and drug components were identified based on the matching scores of tandem mass spectrometry.

### Network pharmacology analysis

#### Acquisition of drug targets

The identified components were imported into the TCSMP database, with OB ≥ 30% and DL ≥ 0.18 as screening criteria. Eligible components were searched for targets using the Swiss Target Prediction with a screening criterion of “Probability > 0”. All targets were subsequently summarized and deduplicated. After standardization through the UniProt database, they were saved as drug targets.

#### Acquisition of PCOS targets

Disease targets for PCOS were retrieved using the Gene Cards database, DisGeNET database, PharmGKB database, TTD database, CTD database, and Drugbank database with the keyword “Polycystic ovary syndrome”. Entries without a Uniprot ID column were excluded. The retrieved results were merged and duplicates were deleted to obtain the PCOS targets.

#### Component- intersection target network diagram

The intersection of all component targets and PCOS targets was taken, and a component-intersection target network diagram was constructed using Cytoscape software. Nodes represent components and intersection targets, and connecting lines represent the interaction relationships between components and intersection targets. The “Analyze Network” plugin was used for topological analysis of components, which were sorted and screened for core components based on degree values.

#### Construction of PPI network diagram and screening of core targets

The intersection targets of ZGP for PCOS treatment were imported into the STRING online platform, with the species limited to “Homo sapiens” and the highest confidence set to 0.400. The protein-protein interaction (PPI) relationships between target proteins were obtained to generate a PPI network diagram. The PPI network diagram was drawn using Cytoscape 3.10.1 software. Topological parameter analysis was performed using CytoNCA. The core targets were sequentially screened based on the conditions that the Betweenness Centrality (BC), Closeness Centrality (CC), and Degree Centrality (DC) were all greater than their respective medians.

#### GO and KEGG enrichment analysis

The intersection targets were imported into the DAVID database for Gene Ontology (GO) enrichment and Kyoto Encyclopedia of Genes and Genomes (KEGG) enrichment analysis. The results were sorted by *P*-value and bubble plots were drawn by https://www.bioinformatics.com.cn.

#### Molecular docking

The format files of obtained core components and core target were imported into Autodock Tools-1.5.7 software. The core components were hydrogenated and set as ligands, while the core targets were dehydrated, hydrogenated, and set as receptors. After setting the docking box and docking parameters, Autodock was run to obtain the binding relationship and binding site between the active molecules and receptor proteins. Finally, PyMOL software was used to visualize the docking results.

### Animal experiment

#### Establishment and intervention of the PCOS model

Female Sprague Dawley rats, aged 8 weeks, SPF grade, with a body weight of (250 ± 20) g, were purchased from and provided by the Hubei Provincial Laboratory Animal Research Center, with a qualification certificate number of SCXK (Hubei) 2020-0018. This experiment was reviewed and approved by the Ethics Committee of the Experimental Animal Center of Hubei University of Chinese Medicine (Ethical Approval Number: HUCMS00306955). The animals were housed in the Experimental Animal Center of Hubei University of Chinese Medicine and underwent adaptive feeding for 7 days before modeling.

The rats were randomly divided into a control (CON) group (*n* = 9) and a PCOS model group (*n* = 24). The PCOS model group was fed a high-fat diet throughout the experiment and received daily gavage with letrozole solution (1 mg/kg) dissolved in 1% carboxymethylcellulose sodium [16]. The CON group was fed with a regular diet and received gavage with an equal volume of solvent for 4 consecutive weeks [17]. After modeling, 3 rats from each group were selected to detect serum hormone levels and observe ovarian tissue pathology. The successfully modeled PCOS group was further divided into a model (MOD) group, a ZGP group, and a metformin (MET) group, with 6 rats in each group. Then, each group was fed with a regular diet from week 5 to week 9. The MOD group and the CON group received gavage with an equal volume of distilled water, the ZGP group received gavage with ZGP suspension, and the metformin group received gavage with metformin aqueous solution for 4 consecutive weeks.

**Fig. 2:**
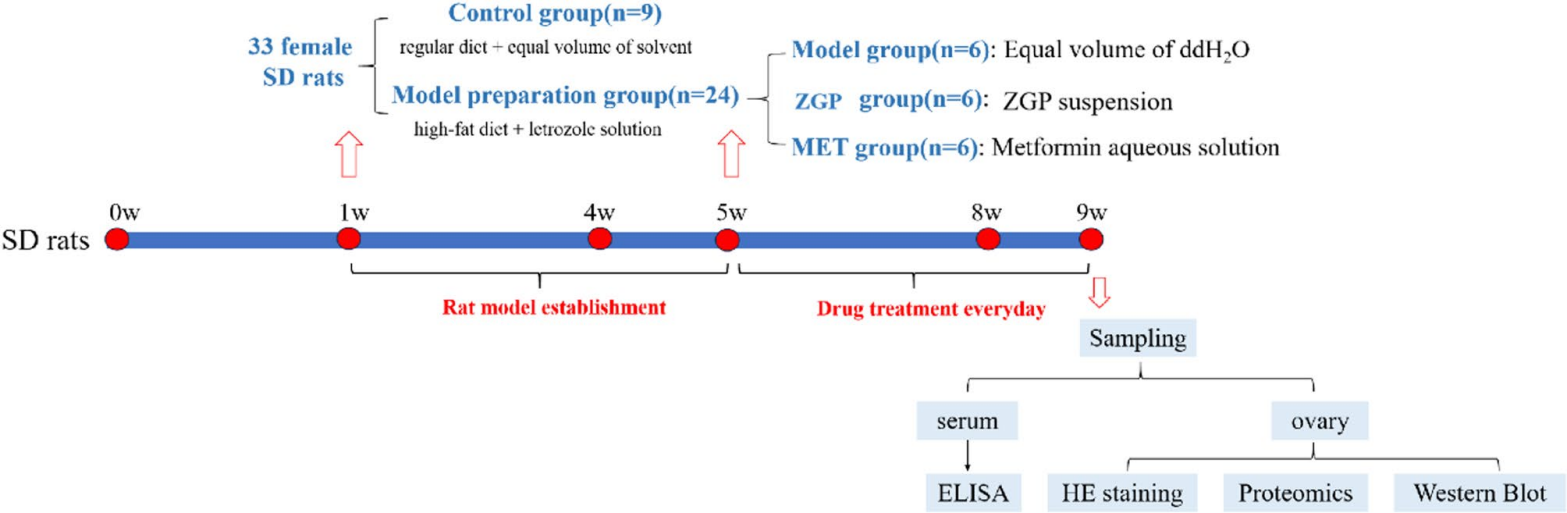
The animal experimental procedure is shown

Sampling was performed after modeling and drug intervention. All rats were weighed and anesthetized with 2% pentobarbital sodium solution (2 mL/kg). After successful anesthesia, venous blood was collected from the heart using a blood collection needle. After allowing the blood to sit for 2 h, it was centrifuged at 3000 rpm for 15 min, and the supernatant was collected for hormone level detection. Both ovaries were harvested and weighed. One ovary was fixed in 4% paraformaldehyde for HE staining, while the other was rapidly frozen in liquid nitrogen and stored at -80 °C for proteomics and molecular biology analysis. The experimental procedure is shown in Fig. 2.

#### Determining serum hormone levels via ELISA

The baseline serum levels of the target hormones, including FSH, LH, E2, AMH, and T, were measured. All operations were performed according to the manufacturer’s instructions.

#### HE staining for histological observation

After fixation in 4% paraformaldehyde, the ovaries underwent dehydration, clearing, wax immersion, embedding, slicing, spreading, and drying. They were then stained with hematoxylin and 1% eosin, mounted, and examined under a microscope to observe ovarian morphology [18].

### Proteomics analysis

#### Quantitative proteomics analysis using 4D-DIA

4D-DIA-based proteomics was used for quantitative analysis of ovarian samples from the CON, MOD, and ZGP groups. The 4D-DIA-based proteomics analysis was conducted by Shanghai Majorbio Bio-pharm Technology Co., Ltd, and the data were analyzed on the Majorbio cloud platform (www.majorbio.com).

#### Western blot

Proteins were extracted from fresh ovaries of each group, lysed, and denatured. After SDS-PAGE electrophoresis, the proteins were transferred to PVDF membranes. After blocking with 5% BSA, the membranes were incubated with primary antibodies diluted 1:1000 overnight, followed by incubation with corresponding secondary antibodies. After washing, the signals were detected using a chemiluminescent reagent, and the signals were collected using a gel imaging system [19]. Finally, the data were analyzed using Image J.

### Data statistics

SPSS V26.0 was used for data analysis, and GraphPad Prism 9.5.1 was used for plotting. All quantitative data are presented as mean ± standard error of the mean (SEM). For data that met the criteria of normal distribution and homogeneity of variance, one-way ANOVA (In one-way ANOVA, means are compared among three or more independent groups) was used for statistical analysis, with a significance threshold set at *P* < 0.05 [20]; otherwise, nonparametric tests were employed.

## Results

### The active ingredients and targets of ZGP

Analysis of the suspension of ZGP using LC-MS revealed that it contains 301 components, with 192 identified in positive ion mode and 109 in negative ion mode. These include 52 flavonoids, 51 terpenes, 23 organic acids and their derivatives, 18 coumarins and their derivatives, 16 carbohydrates, 14 amino acids and their analogs, 9 phenylpropanoids, 6 alcohols, and 7 phenols.

After screening the components of ZGP through the TCSMP database, 31 components were obtained, including 25 flavonoids, 1 phenol, 1 quinone, 1 organic acid and its derivative, 1 steroid and its derivative, 1 terpene, and 1 other. See Table 1 and Fig.S1A-B. A total of 2419 targets were retrieved using Swiss Target Prediction, and 585 targets were retrieved using TCSMP, resulting in a total of 2876 targets. After removing duplicates, 602 targets remained.

### Disease targets and intersection targets

**Fig. 3:**
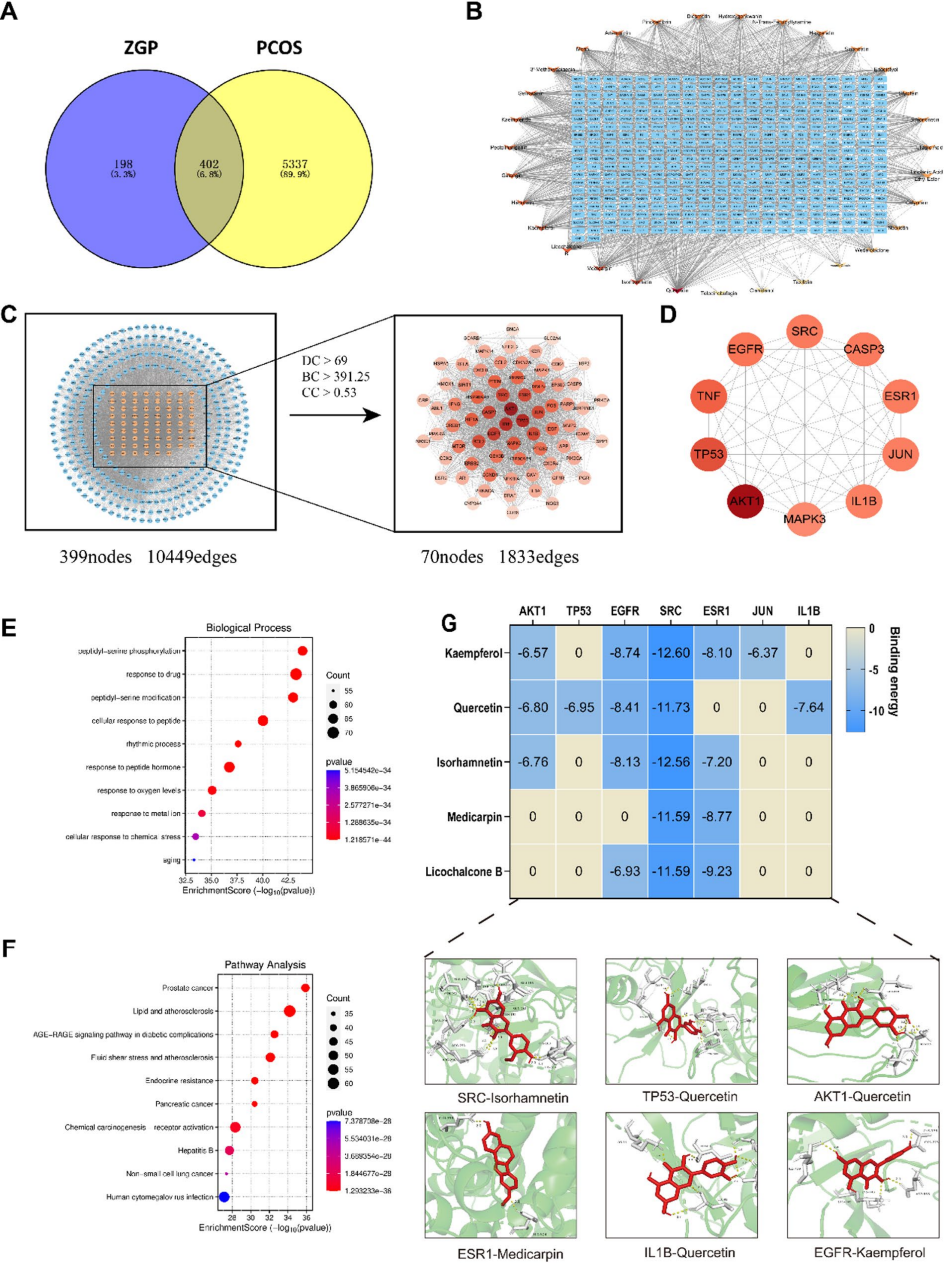
Network pharmacology predicting the potential mechanism of ZGP in PCOS. (**A**) Illustrate Venn diagram, (**B**) ”component-intersection target” network diagram, (**C**) PPI analysis, (**D**) shows top 9 hub genes PPI network, (**E**) represent the GO biological process, (**F**) represent the KEGG pathways, and (**G**) binding energy of molecular docking and representative images

A total of 5739 PCOS targets were obtained, with 402 intersections with drug targets (Fig. 3A). The constructed “component-intersection target” network diagram contains 433 nodes and 1965 connecting lines (Fig. 3B). The top-ranked components by Degree value are quercetin, kaempferol, isorhamnetin, medicarpin, and Licochalcone B, as shown in Table 2 and Fig.S2. PPI analysis of the intersection targets resulted in a diagram with 399 nodes and 10,449 connecting lines (Fig. 3C). The top 9 targets ranked by DC, BC, and CC values are AKT1, TP53, TNF, EGFR, SRC, CASP3, ESR1, JUN, IL1B (Fig. 3D), with detailed information provided in Table 3.

### GO enrichment and KEGG enrichment analysis

GO enrichment results showed that the intersection targets are involved in biological processes such as response to drug, peptidyl-serine phosphorylation, peptidyl-serine modification, cellular response to peptide, rhythmic process, and response to peptide hormone (Fig. 3E). The involved molecular structures include membrane raft, membrane microdomain, membrane region, vesicle lumen, protein kinase complex, plasma membrane raft (Fig. S3A). The involved molecular functions (MF) include protein serine/threonine kinase activity, protein tyrosine kinase activity, transmembrane receptor protein tyrosine kinase activity, transmembrane receptor protein kinase activity, drug binding, and kinase regulator activity (Fig. S3B). KEGG pathway enrichment included pathways such as Prostate cancer, Lipid and atherosclerosis, AGE-RAGE signaling pathway in diabetic complications, Fluid shear stress and atherosclerosis, Pancreatic cancer, Endocrine resistance, and Chemical carcinogenesis-receptor signaling pathway (Fig. 3F).

### Molecular docking results

Molecular docking results showed that the binding energies between core components and core targets were all less than − 5 kcal/mol (Figs. 3G), indicating that the active components obtained from ZGP can stably dock with the targets. Among them, kaempferol, quercetin, and isorhamnetin have good binding ability with core targets and may play a potential role in the treatment of PCOS.

**Table 1:** Components were obtained by screening the components of ZGP through the TCSMP database

No.	Metabolite	m/z	Retention time	Mode	Adducts	Formula	PPM	Classification
neg-1	Cianidanol	289.07	4.32	neg	M-H	C15H14O6	-3.81	Flavonoids
neg-2	Kaempferide	299.05	6.71	neg	M-H	C16H12O6	-4.12	Flavonoids
neg-3	Pectolinarigenin	313.07	7.05	neg	M-H	C17H14O6	-4.24	Flavonoids
neg-4	Calycosin	283.06	8.00	neg	M-H	C16H12O5	-4.24	Flavonoids
neg-5	Wedelolactone	313.03	8.56	neg	M-H	C16H10O7	-3.99	Flavonoids
neg-6	Medicarpin	269.08	9.47	neg	M-H	C16H14O4	-4.35	Flavonoids
neg-7	Pinocembrin	255.07	10.35	neg	M-H	C15H12O4	-4.86	Flavonoids
neg-8	Galangin	269.04	10.49	neg	M-H	C15H10O5	-4.39	Flavonoids
neg-9	Diosmetin	299.05	8.20	neg	M-H	C16H12O6	-4.28	Flavonoids
neg-10	Isorhamnetin	315.05	7.37	neg	M-H	C16H12O7	-3.92	Flavonoids
neg-11	3’-Methoxyapigenin	299.05	7.16	neg	M-H	C16H12O6	-4.32	Flavonoids
neg-12	Kaempferol	285.04	7.07	neg	M-H	C15H10O6	-4.54	Flavonoids
neg-13	Quercetin	301.03	5.81	neg	M-H	C15H10O7	-4.54	Flavonoids
neg-14	Genkwanin	283.06	5.67	neg	M-H	C16H12O5	-4.45	Flavonoids
neg-15	N-trans-Feruloyltyramine	312.12	5.47	neg	M-H	C18H19NO4	-3.88	Phenols
neg-16	Ellagic acid	301.00	3.98	neg	M-H	C14H6O8	-4.24	Organic acids and their derivatives

**Table 2:** The top components are ranked by degree value

Compound	DC	BC	CC
Kaempferide	163	0.075	0.405
Quercetin	127	0.208	0.423
Isorhamnetin	94	0.050	0.398
Medicarpin	91	0.133	0.396
Licochalcone B	86	0.099	0.392

**Table 3:** The top 9 targets are ranked by DC, BC, and CC values

Gene	DC	BC	CC
AKT1	253	8617.024	0.729
TNF	222	6208.172	0.689
TP53	228	6344.559	0.696
EGFR	205	4238.571	0.667
SRC	197	7043.518	0.655
IL1B	191	3433.456	0.654
ESR1	195	5136.895	0.657
JUN	192	2891.439	0.654
MAPK3	178	3389.688	0.639

### General biological characteristics and histomorphological analysis

**Fig. 4:**
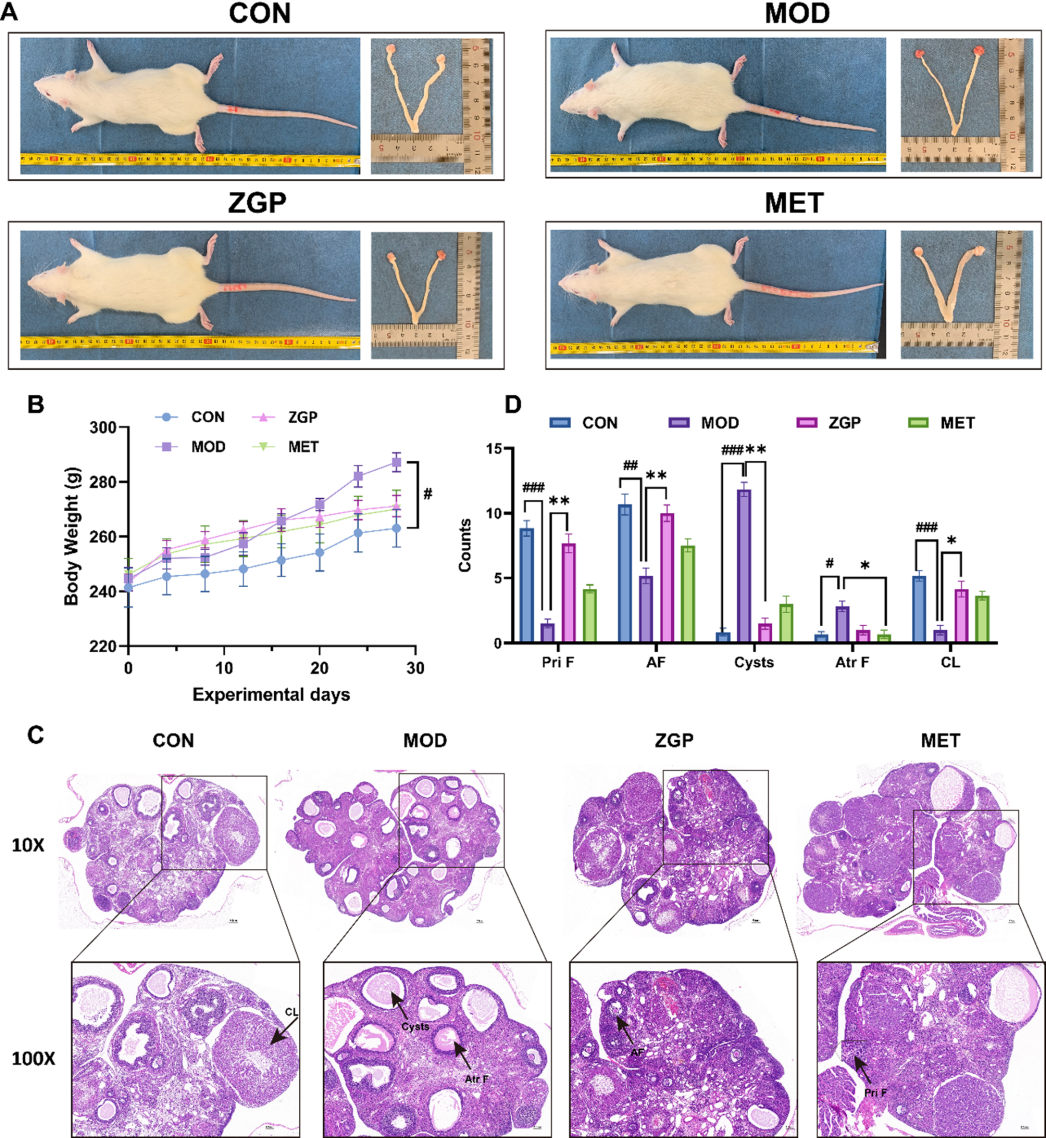
ZGP ameliorated growth and ovarian tissue damage in PCOS rats(*n* = 6). (**A**) Rat whole body, ovary and uterus gross appearance. (**B**) Weight changes in rats. (**C**) Ovarian representative pathological images, and (**D**) Follicle count. CL, corpus luteum; Cysts, follicular cysts; Atr F, atresia follicles; AF, antral follicles; Pri F, primary follicle. #*P* < 0.05, ##*P* < 0.01, ###*P* < 0.001 compared to the CON; *P < 0.05, **P < 0.01 compared to the MOD

Compared with the CON group, although there was no significant difference in appearance, the MOD group showed significantly increased body weight (*P <* 0.05) and a marked increase in the number of ovarian vesicles visible macroscopically. Compared with the MOD group, the ZGP group and the metformin group showed a decreasing trend in body weight and a significant reduction in the number of ovarian vesicles (Figs. 4A, B). HE staining results (Figs. 4C, D) showed normal growth and development of follicles at various stages in the ovarian sections of the CON group. The MOD group showed significant vesicle accumulation, with quantitative data indicating a significant increase in the number of vesicular ovaries (*P <* 0.001), a trend of increased atretic follicles (*P <* 0.05) and a decrease in corpus luteum count (*P <* 0.001). The ZGP group showed a significant reduction in vesicle count (*P <* 0.01), a significant decrease in atretic follicle count compared with the MOD group (*P <* 0.05), and an increase in corpus luteum count (*P <* 0.05).

### Serum sex hormone levels

**Fig. 5:**
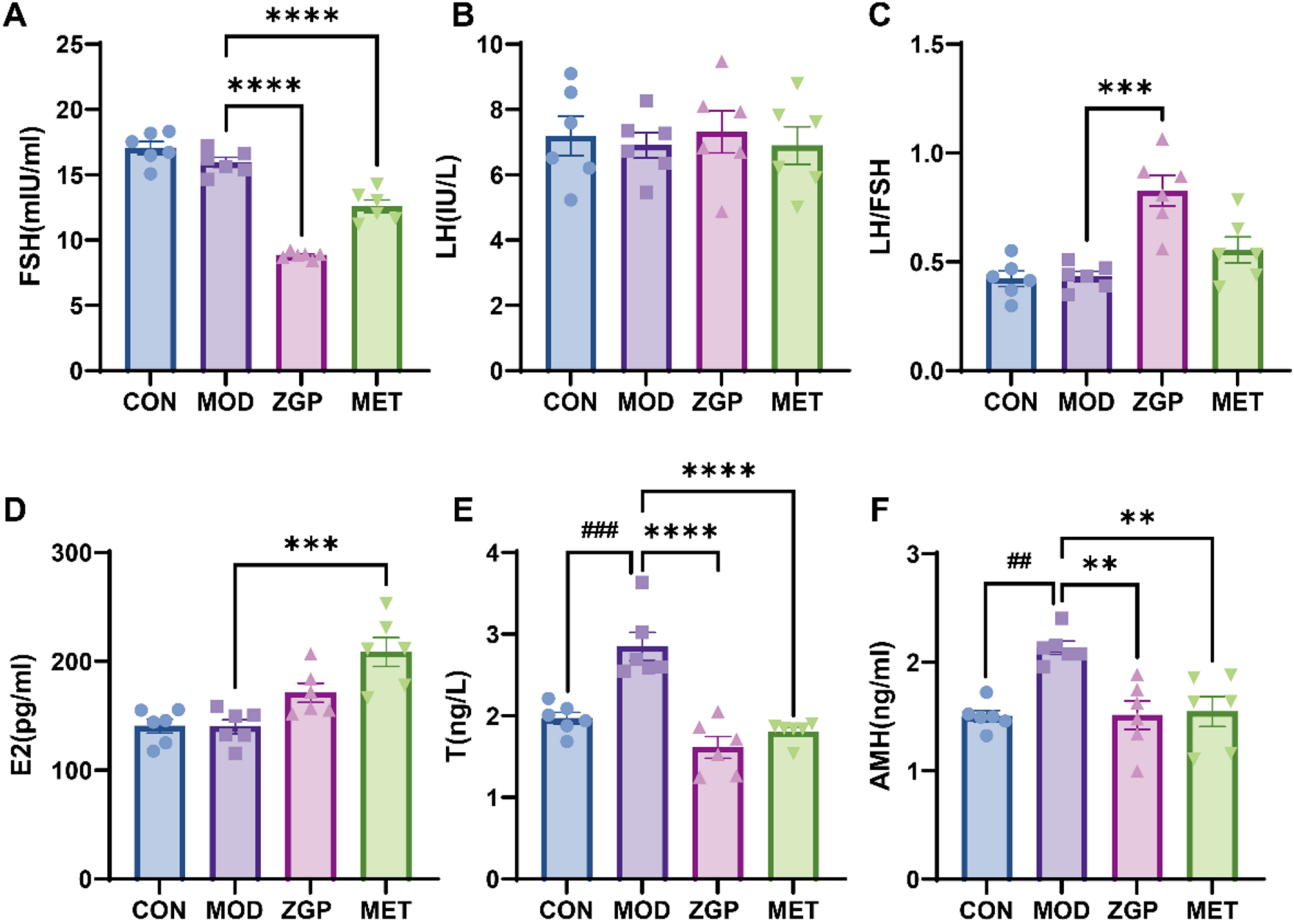
ZGP improved serum sex hormone levels in PCOS rats(*n* = 6). ^##^*P* < 0.01, ^###^*P* < 0.001 compared to the CON; ^**^P < 0.01, ^***^P < 0.001, ^****^P < 0.0001 compared to the MOD

As shown in Fig. 5, compared with the CON group, there were no significant differences in FSH, LH, E2, and LH/FSH levels in the MOD group, but T and AMH levels were significantly increased (*P <* 0.001 and *P <* 0.01, respectively). Compared to the MOD group, FSH levels of ZGP group were significantly decreased (*P <* 0.0001), LH levels showed no significant difference, the LH/FSH ratio was significantly increased (*P <* 0.001), T levels were significantly decreased (*P <* 0.0001), and AMH levels were significantly reduced (*P <* 0.01). There was no significant difference between the ZGP group and the metformin group.

### Proteomics analysis of the potential mechanism

**Fig. 6:**
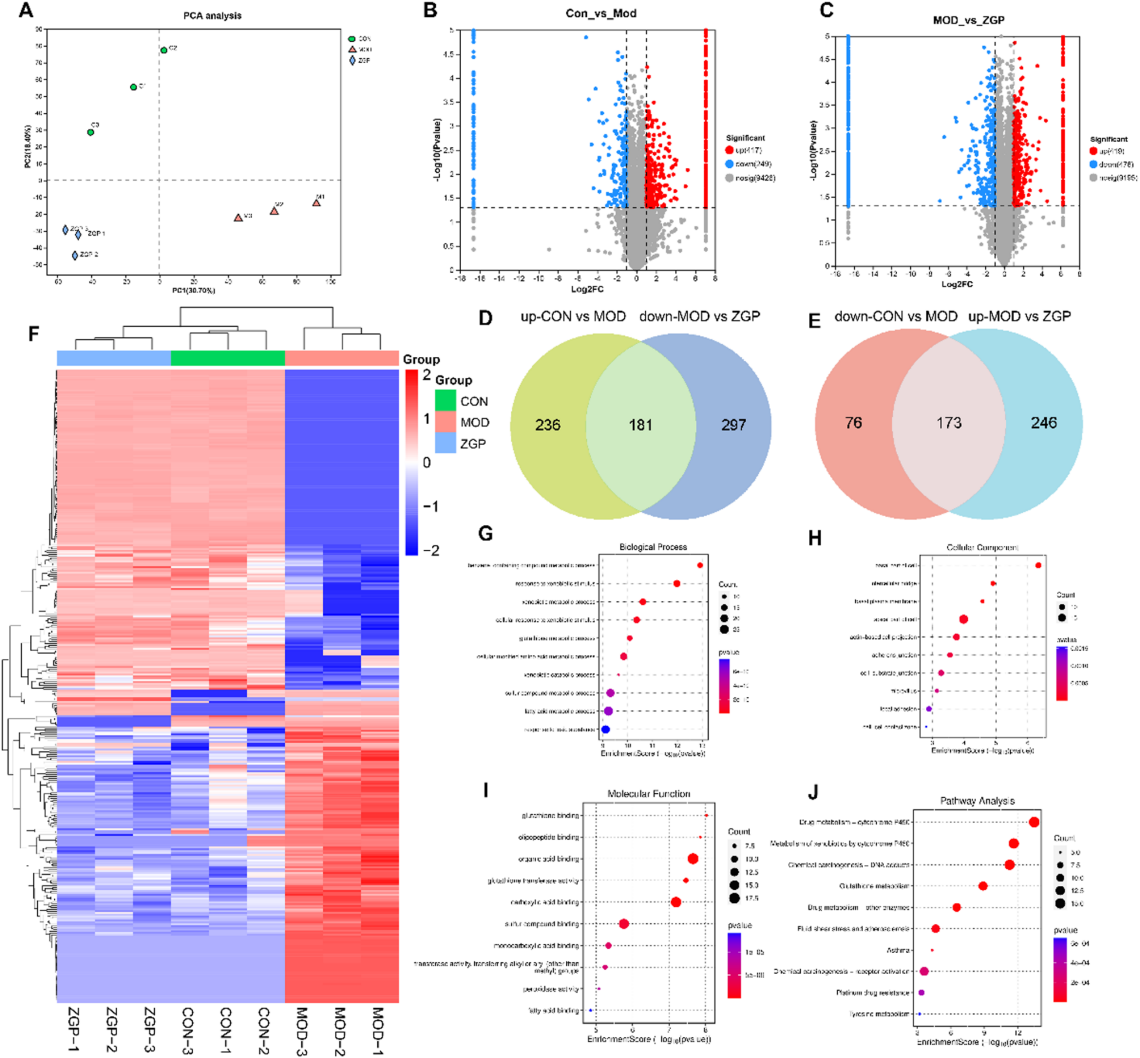
Proteomics analysis of the potential mechanism of ZGP in improving PCOS. (**A**) PCA analysis. (**B-C**) Volcano plot of CON and MOD, MOD and ZGP. (**D-E**) Venn diagram of ZGP down-regulated and up-regulated differential proteins. (**F**) Heat map of differential proteins. (**G-I**) Represent the GO biological process, Cellular Component and Molecular Function. (**J**) represent the KEGG pathways

4D-DIA-based proteomics analysis was used to determine differences in protein types between groups. The relationship between peptide count and peptide length is shown in Fig.S4A-B. The cluster heatmap demonstrated good reproducibility between the two replicate samples (Fig.S4C). According to the PCA plot, the CON group and the MOD group were clearly separated, with good intragroup sample clustering. The ZGP group was also clearly separated from the MOD group, indicating a reversal effect of ZGP to some extent (Fig. 6A). Data processing of proteins common to the CON and MOD groups revealed 249 downregulated and 417 upregulated proteins (Fig. 6B). Data processing of proteins common to the ZGP and MOD groups revealed 478 downregulated and 419 upregulated proteins (Fig. 6C). To explore the pharmacological effects of ZGP, the differentially expressed proteins (DEPs) with reversed expression trends after ZGP treatment were identified, resulting in 354 proteins with significant reversal, including 173 upregulated (Fig. 6D) and 181 downregulated (Fig. 6E). A cluster heatmap of the 354 proteins clearly showed stratified DEPs clustering among the three groups (Fig. 6F). Detailed information on the 354 proteins is provided in Table S2.

To determine the relevant biological processes involved in the therapeutic effect of ZGP, GO and KEGG enrichment analyses were performed on the 354 proteins. Specifically, 55 BP terms, 56 CC terms, 99 MF terms, and 27 KEGG pathway terms were significantly enriched, respectively. BP results showed that benzene-containing compound metabolic process, response to xenobiotic stimulus, and xenobiotic metabolic process (Fig. 6G). CC results indicated their involvement in the basal part of cell, intercellular bridge, basal plasma membrane, and apical part of cell (Fig. 6H). MF analysis correlated them with glutathione binding, oligopeptide binding, organic acid binding, and glutathione transferase activity (Fig. 6I). The KEGG pathways implicated these proteins primarily in Drug metabolism-cytochrome P450, Metabolism of xenobiotics by cytochrome P450, Chemical carcinogenesis - DNA adducts, Glutathione metabolism, and Drug metabolism -other enzymes (Fig. 6J).

### Combined analysis of network pharmacology and proteomics analysis

**Fig. 7:**
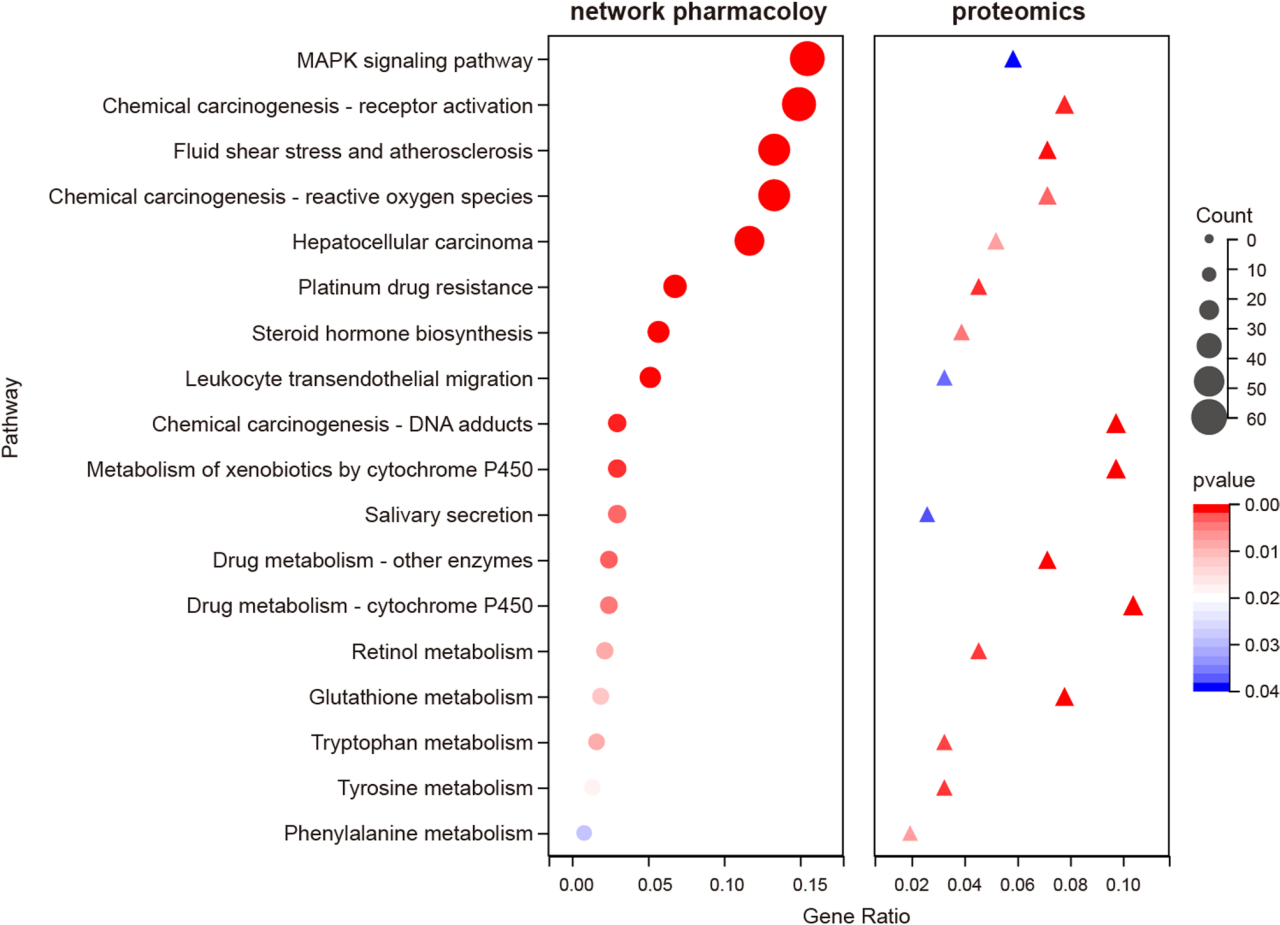
Bubble maps of common differential pathways in proteomics and network pharmacology

**Fig. 8:**
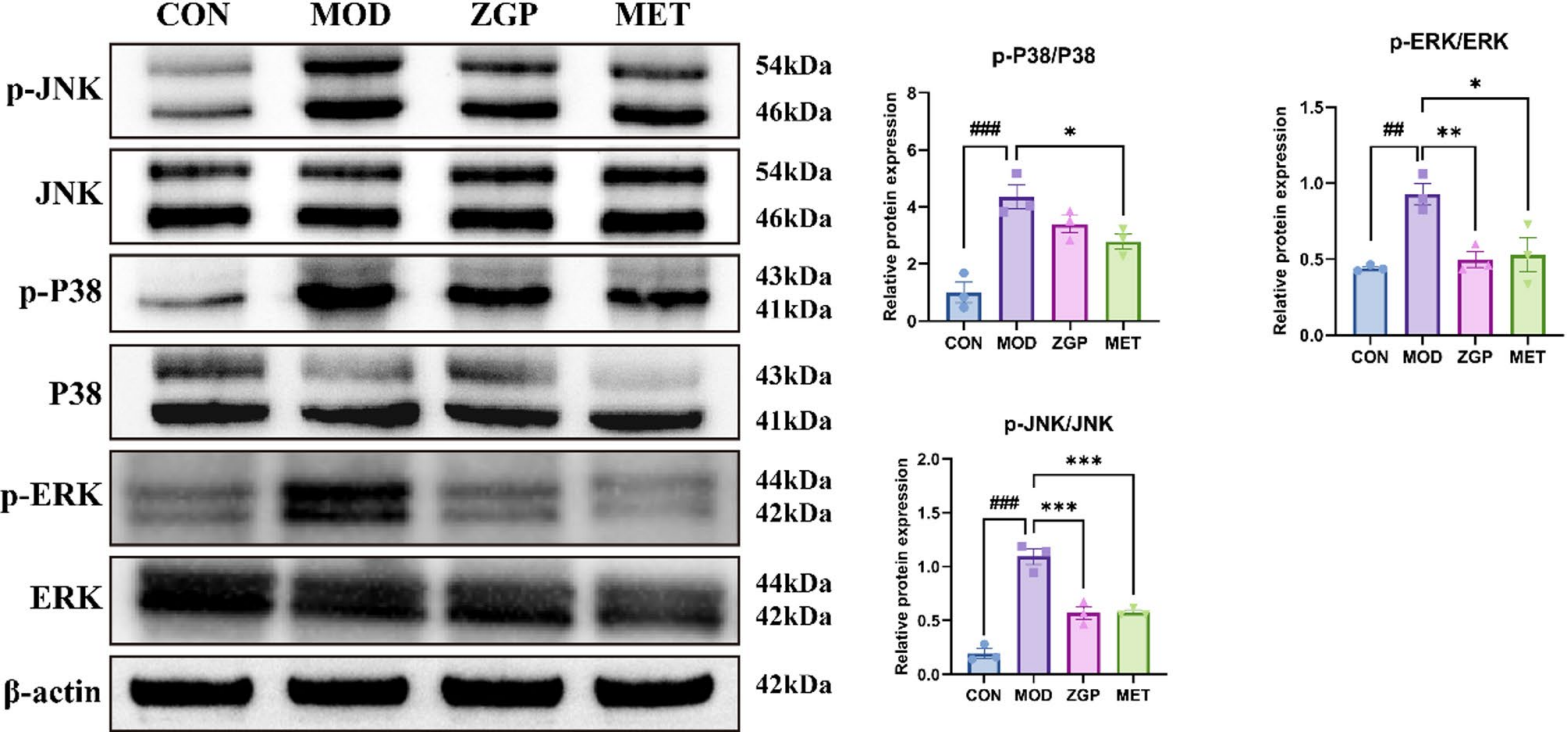
Expression levels of MAPK pathway-related proteins. ^##^*P* < 0.01, ^###^*P* < 0.001 compared to the CON; ^*^P < 0.05, ^**^P < 0.01, ^***^P < 0.001 compared to the MOD

A combined analysis of the pathway results from network pharmacology and proteomics was conducted to further explore the mechanism of action of ZGP. There were 10 common differential pathways between network pharmacology and proteomics, including MAPK signaling pathway, steroid hormone biosynthesis, and PPAR signaling pathway (Fig. 7). WB analysis revealed that compared with the CON group, the expression levels of p-ERK1/2 and p-JNK were significantly increased in the MOD group (*P <* 0.05). However, after treatment with ZGP, their expression levels tended to normalize (*P <* 0.05) (Fig. 8). Although there was a trend of reversal in P38 MAPK expression, it did not reach statistical significance (*P* > 0.05).

## Discussion

PCOS is a complex gynecological disorder characterized by irregular menstrual cycles, abnormal ovulation, difficulties in conception, and the formation of multiple cysts within the ovaries [21]. Despite the lack of a definitive etiology, research suggests that PCOS may be associated with metabolic abnormalities, dysfunction of the HPO axis, imbalances in nutrition, and the use of certain medications [22]. Various pharmacological treatment options have been proposed for PCOS. Among them, oral contraceptives are one of the most commonly used treatments, with mechanisms that include reducing free testosterone levels in the blood and inhibiting FSH secretion, thereby exerting therapeutic effects [23]. Additionally, clomiphene citrate and letrozole are also widely used in the treatment of PCOS [24, 25]. However, these medications have drawbacks such as adverse reactions, low patient compliance with long-term medication, low efficacy, and contraindications in some cases [26]. Therefore, an effective therapy for the radical treatment of PCOS has not yet been found [27, 28].

TCM has a long history in the treatment of gynecological disorders and infertility. Research results indicate that many Chinese herbal medicines have therapeutic effects on various aspects of PCOS, including menstrual and ovulation dysfunction, obesity, IR, hyperinsulinemia, lipid metabolic dysfunction, hirsutism, and other androgen excess-related conditions [29, 30].

ZGP is a TCM formula consisting of eight herbs, including Shudihuang [the dried root tuber of R*ehmannia glutinosa (Gaertn.) DC.*], Tusizi (the dried ripe seed of *Cuscuta chinensis Lam.*), Niuxi (the dried root of *Achyranthes bidentata Blume*), Guibanjiao (*Colla carapacis et plastri testudinis*), Lujiaojiao (*Pulvis Cornu Cervi*), Shanyao (the dried rhizome of *Dioscorea oppositifolia L.*), Shanzhuyu (the dried ripe sarcocarp of *Cornus officinalis Siebold & Zucc.*), and Gouqizi (the dried ripe fruit of *Lycium barbarum L.*).

As a classic TCM formula, ZGP has the effects of nourishing the kidney, promoting the production of essence, and benefiting the marrow [12]. Its efficacy lies in nourishing kidney yin and maintaining the balance of yin and yang in the body, thereby improving reproductive health. Previous studies have thoroughly revealed the efficacy of ZGP from different perspectives. Firstly, ZGP has been shown to have protective effects against ovarian aging by upregulating the Notch signaling pathway to maintain the characteristics and functions of ovarian stem cells (OSCs) [31]. Secondly, another study indicated that ZGP alleviates the symptoms of perimenopausal syndrome in mice by regulating apoptosis and improves their quality of life [32]. Li Zhuang’s research delved into the mechanism of ZGP against cyclophosphamide-induced ovarian aging. Li found that ZGP counteracts ovarian aging by restoring ovarian function, alleviating oxidative stress in aged OSCs, promoting OSCs proliferation, and restoring their stem cell characteristics. In this process, ZGP may play a key role by regulating the Nrf2/HO-1 pathway. The Nrf2/HO-1 pathway is one of the important mechanisms of intracellular antioxidant stress, which can mitigate oxidative stress damage by regulating the expression of antioxidant enzymes [33]. By activating this pathway, ZGP helps protect the ovaries from damage caused by harmful factors such as cyclophosphamide [13]. Furthermore, researchers discovered the potential of ZGP to promote the recovery of ovarian function in prematurely senescent rats. By comparing ovarian images of prematurely senescent rats before and after ZGP treatment, researchers found that ZGP significantly improves ovarian morphological structure and promotes follicular development. This discovery further broadens our understanding of the mechanism of action of ZGP [34].

Through combined analysis, we identified the MAPK signaling pathway as a key differential pathway in the treatment of PCOS with ZGP. The MAPK signaling pathway is a major regulatory module for various cellular processes such as cell proliferation, differentiation, and stress responses [35]. In ovarian tissue, the expression of a large number of genes is significantly associated with the MAPK signaling pathway [36]. At least four different types of MAPK exist in mammals: extracellular signal-related kinase (Erk)-1/2, Jun amino-terminal kinase (Jnk1/2/3), p38 proteins (p38alpha/beta/gamma/delta), and Erk5 [37]. Studies have shown that the expression of Erk1/2 (MAPK3/1) in ovarian granulosa cells is crucial for maintaining female fertility, as it regulates LH-induced oocyte maturation during meiosis, ovulation, and luteinization stages [38]. Additionally, Erk1 and Erk2 are expressed in all mammalian tissues and are considered important regulators of cell proliferation, differentiation, and oocyte maturation in vitro [39]. However, genetic studies have shown that the deletion of the Erk1 gene has a relatively small impact on fertility and embryonic survival rates [40], while mutations in the Erk2 gene are lethal to mouse embryos [41].

In this study, abnormal protein expression in the MAPK signaling pathway was observed, and the expression levels of proteins in this pathway were modulated after ZGP treatment, suggesting that ZGP may exert therapeutic effects on PCOS by regulating the MAPK signaling pathway.

## Conclusion

In summary, this study preliminarily indicates via network pharmacology and proteomics that ZGP may ameliorate the hormonal disturbances of PCOS by suppressing the phosphorylation of JNK/ERK, key nodes within the MAPK pathway. Nevertheless, the relative therapeutic advantage of ZGP and the cascade relationship between its upstream triggers and downstream effector molecules remain to be rigorously validated.

## Supplementary Information

Below is the link to the electronic supplementary material.

Supplementary Material 1

## Data Availability

No datasets were generated or analysed during the current study.

## Abbreviations

PCOS: Polycystic ovary syndrome
ZGP: Zuogui pill
LC-MS: Liquid chromatography-mass spectrometry
HE: Hematoxylin and eosin
WB: Western blot
MAPK: Mitogen-activated protein kinase
LH: Luteinizing hormone
IR: Insulin resistance
TCM: Traditional Chinese medicine
HPO: Hypothalamic-pituitary-ovarian
FSH: Follicle-stimulating hormone
AMH: Anti-Müllerian hormone
QC: Quality control
UHPLC: Ultra-high-performance liquid chromatography
MS: Mass spectrometry
PPI: Protein-protein interaction
BC: Betweenness centrality
CC: Closeness centrality
DC: Degree centrality
GO: Gene ontology
KEGG: Kyoto encyclopedia of genes and genomes
CON: Control
MOD: Model
MET: Metformin
SEM: Standard error of the mean
MF: Molecular functions
DEPs: Differentially expressed proteins
OSCs: Ovarian stem cells
