# Supplementary material for: Mechanism of Zuogui pill in the treatment of polycystic ovary syndrome based on LC-MS and proteomics
Source: J Ovarian Res. 2025 Sep 26;18:205. doi: 10.1186/s13048-025-01802-3 (PMC12465971; doi:10.1186/s13048-025-01802-3)
Supplement: Supplementary file 1 — Supplementary Material 1 [file 13048_2025_1802_MOESM1_ESM.docx]

Suppl. figure 1: p-ERK


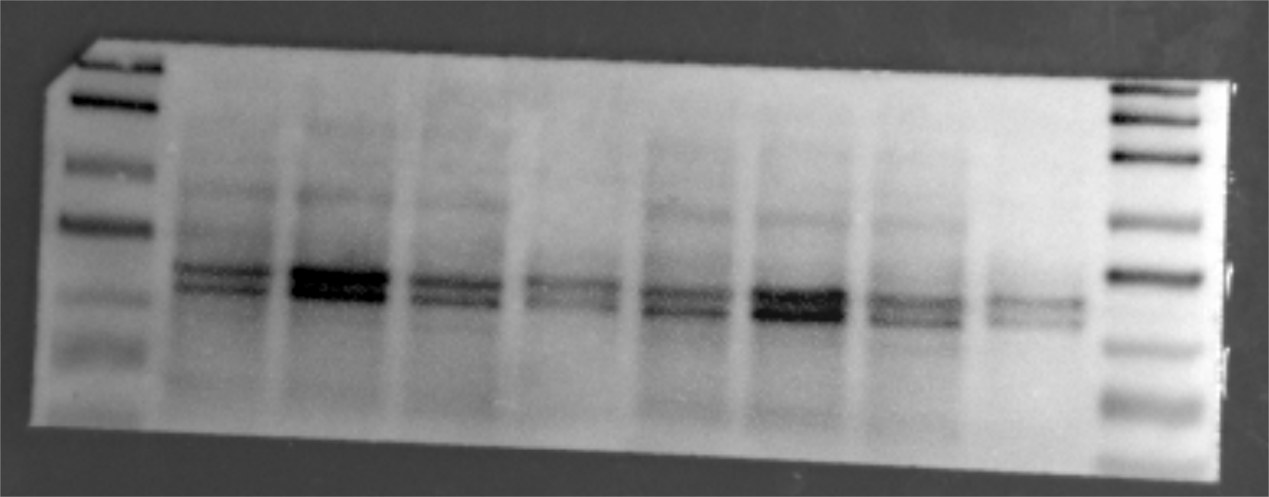


170kD

130kD

70kD

55kD

45kD

35kD

100kD

25kD

MET

ZGP

MOD

CON

②

CON

MOD

ZGP

MET

①


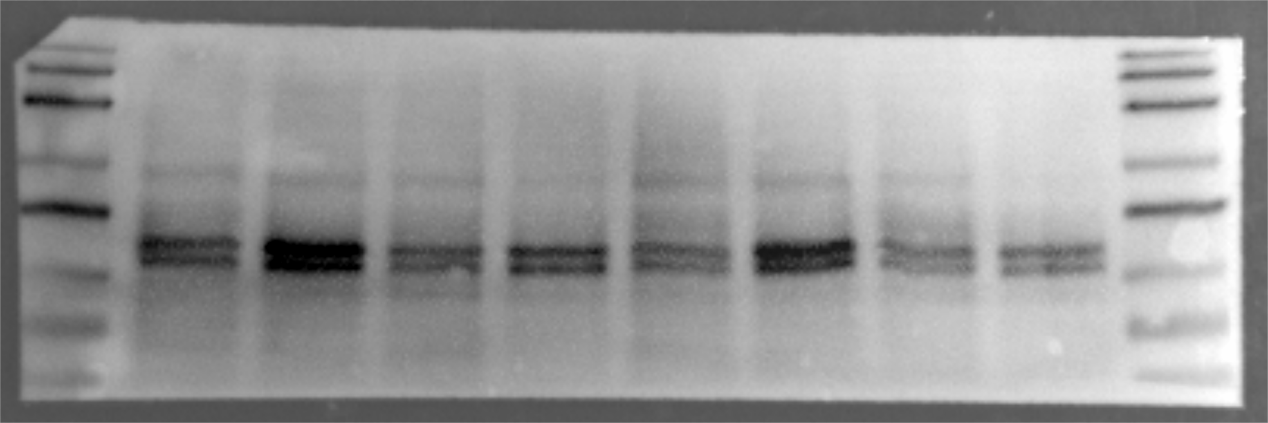


170kD

70kD

55kD

45kD

35kD

100kD

25kD

③

MOD

MET

ZGP

CON

Suppl. figure 2: ERK


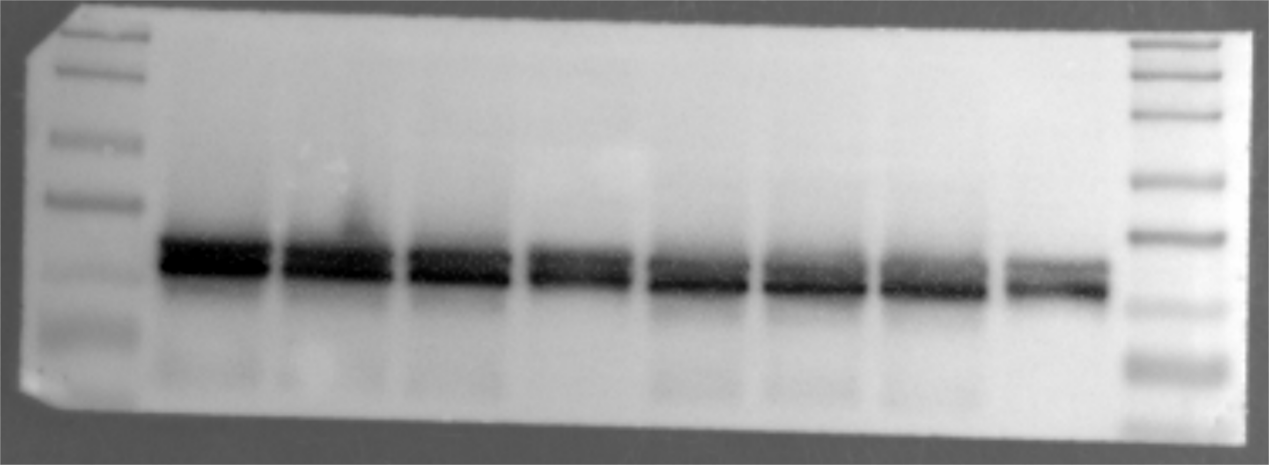


170kD

130kD

70kD

55kD

45kD

35kD

100kD

25kD

CON

MOD

ZGP

MET

①

CON

MOD

ZGP

MET

②


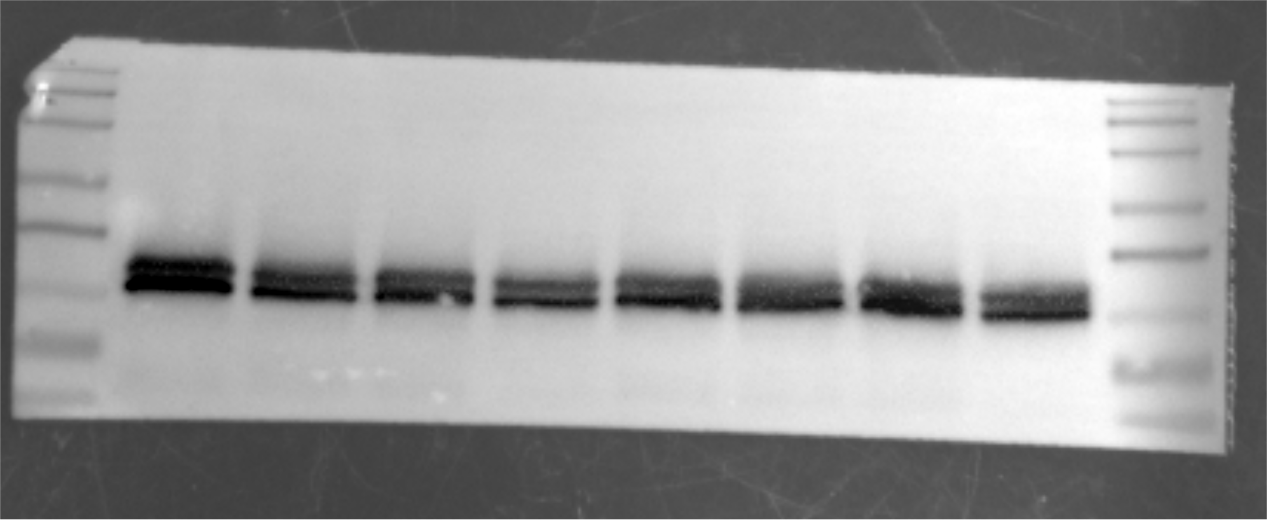


170kD

130kD

70kD

55kD

45kD

35kD

100kD

25kD

CON

MOD

ZGP

MET

③

Suppl. figure 3: JNK


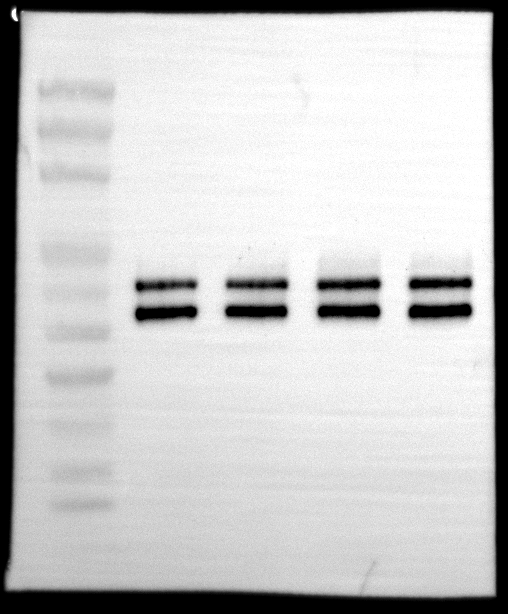


250kD

150kD

70kD

50kD

40kD

35kD

100kD

25kD

20kD

15kD

CON

MOD

ZGP

MET

①


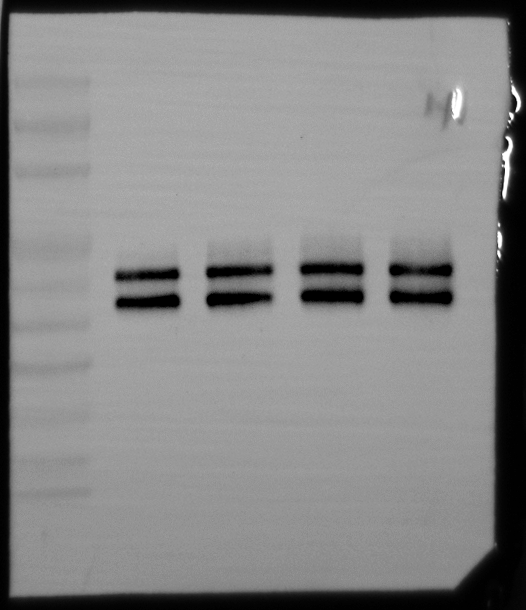


250kD

150kD

70kD

50kD

40kD

35kD

100kD

25kD

20kD

15kD

CON

MOD

ZGP

MET

②


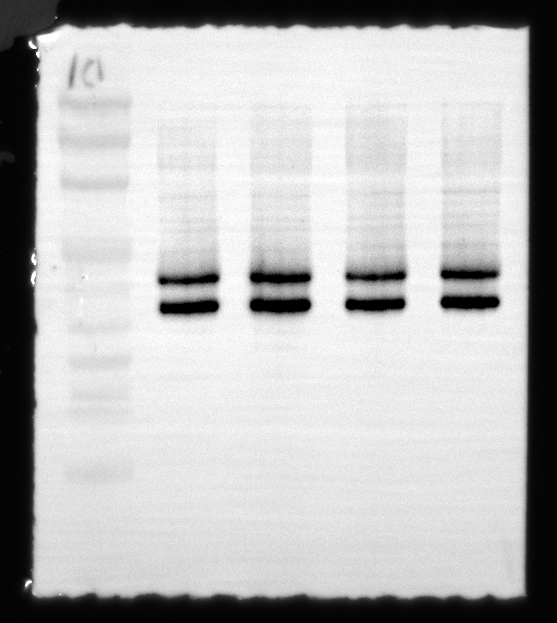


250kD

150kD

70kD

50kD

40kD

35kD

100kD

25kD

20kD

15kD

CON

MOD

ZGP

MET

③

Suppl. figure 4: p38


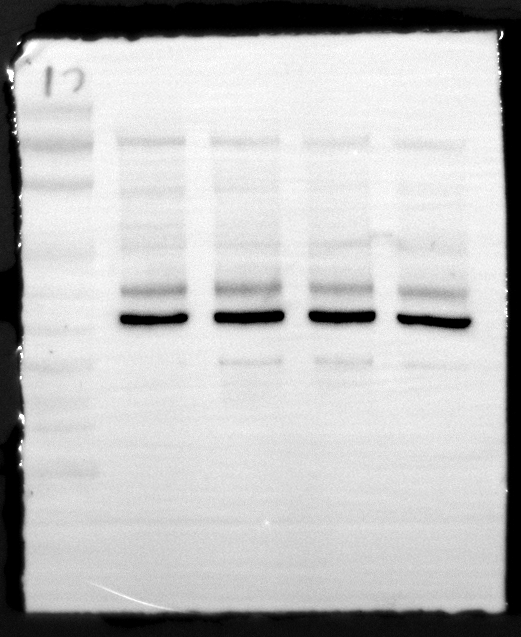


250kD

150kD

70kD

50kD

40kD

35kD

100kD

25kD

20kD

15kD

CON

MOD

ZGP

MET

①


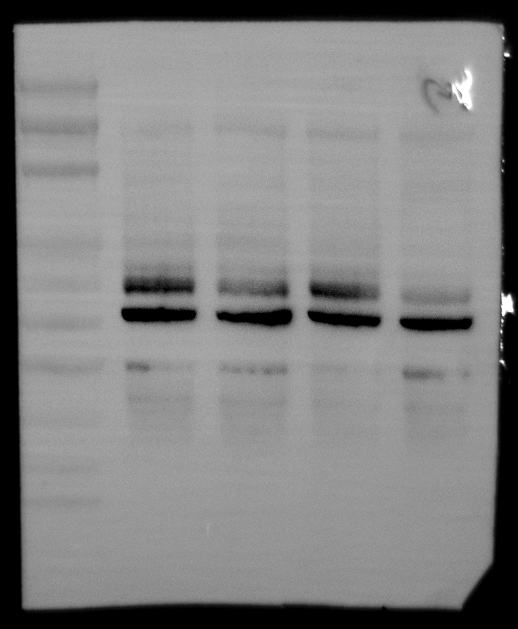


250kD

150kD

70kD

50kD

40kD

35kD

100kD

25kD

20kD

15kD

CON

MOD

ZGP

MET

②


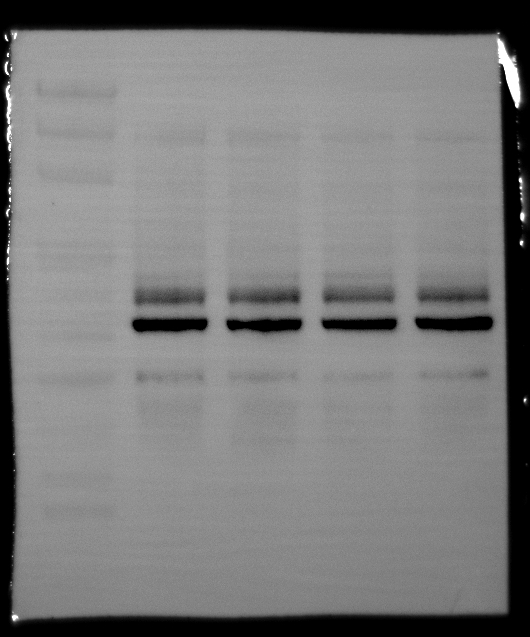


250kD

150kD

70kD

50kD

40kD

35kD

100kD

25kD

20kD

15kD

CON

MOD

ZGP

MET

③

Suppl. figure 5: P-JNK


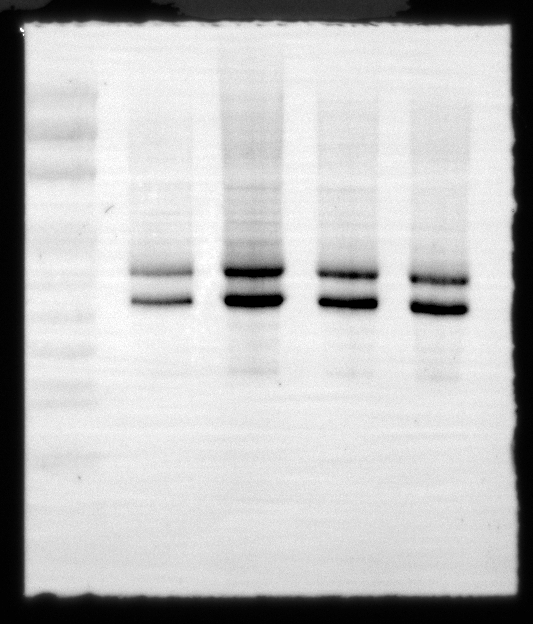


250kD

150kD

70kD

50kD

40kD

35kD

100kD

25kD

20kD

15kD

CON

MOD

ZGP

MET

①


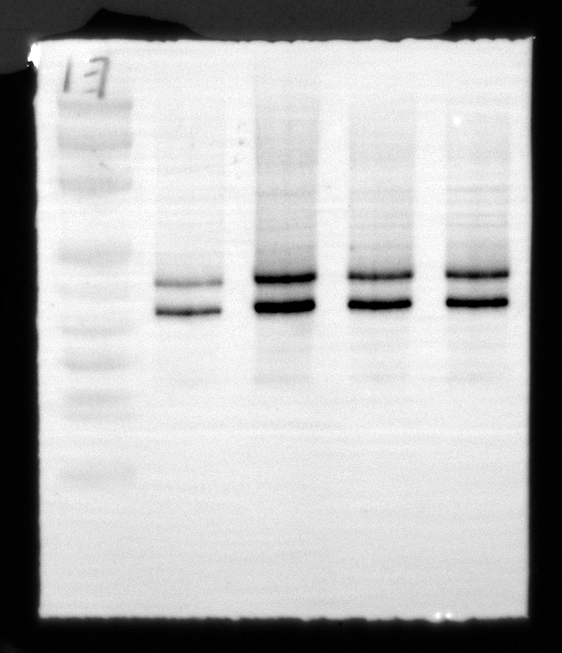


250kD

150kD

70kD

50kD

40kD

35kD

100kD

25kD

20kD

15kD

CON

MOD

ZGP

MET

②


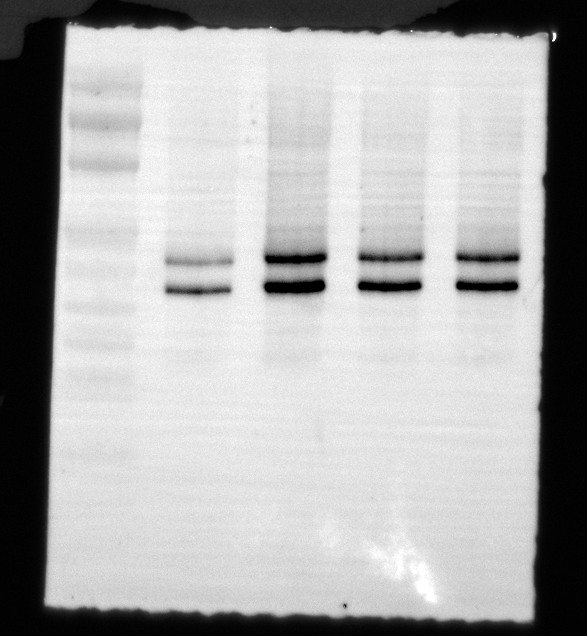


250kD

150kD

70kD

50kD

40kD

35kD

100kD

25kD

20kD

15kD

CON

MOD

ZGP

MET

③

Suppl. figure 6: p-p38


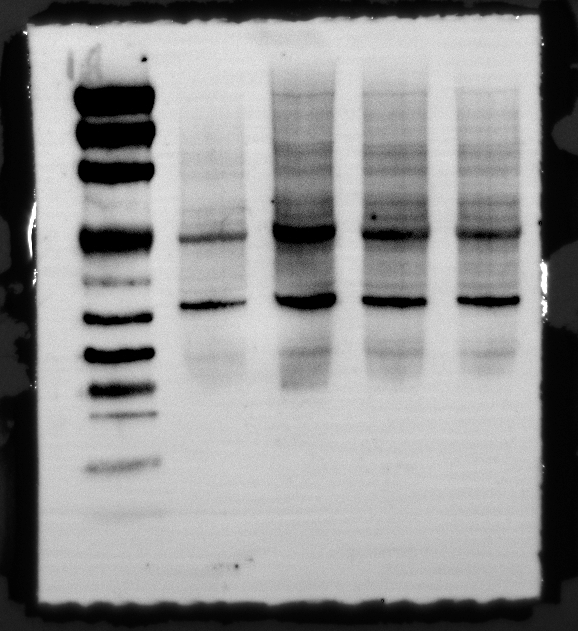


250kD

150kD

70kD

50kD

40kD

35kD

100kD

25kD

20kD

15kD

CON

MOD

ZGP

MET

①


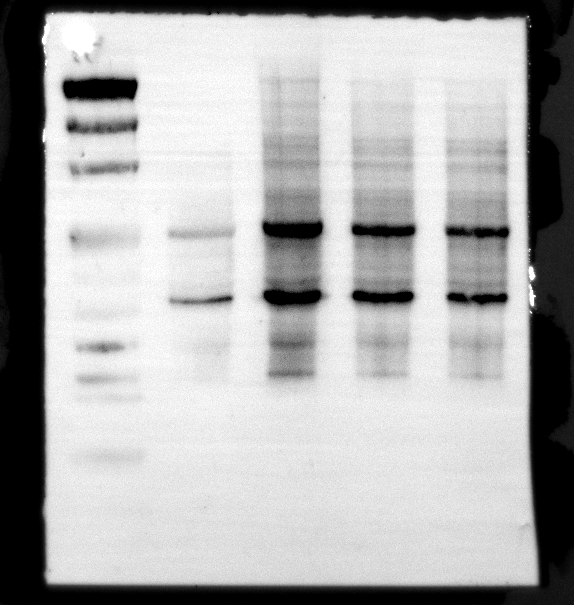


250kD

150kD

70kD

50kD

40kD

35kD

100kD

25kD

20kD

15kD

CON

MOD

ZGP

MET

②


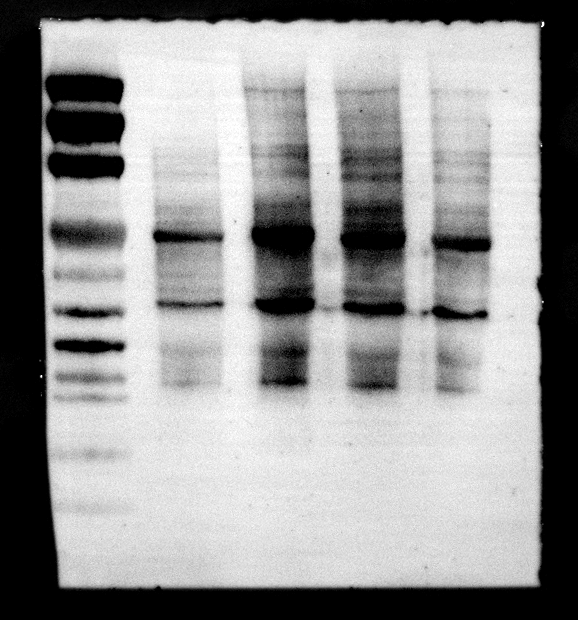


250kD

150kD

70kD

50kD

40kD

35kD

100kD

25kD

20kD

15kD

CON

MOD

ZGP

MET

③

Suppl. figure 7: β-Actin


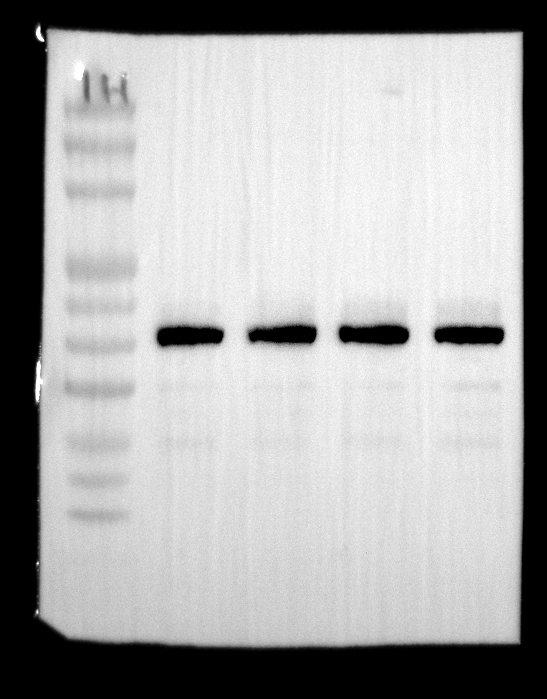


250kD

150kD

70kD

50kD

40kD

35kD

100kD

25kD

20kD

15kD

CON

MOD

ZGP

MET

①


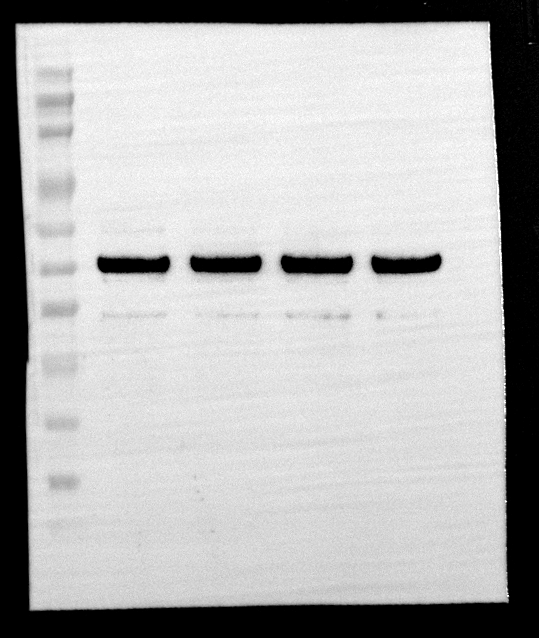


250kD

150kD

70kD

50kD

40kD

35kD

100kD

25kD

20kD

15kD

CON

MOD

ZGP

MET

②


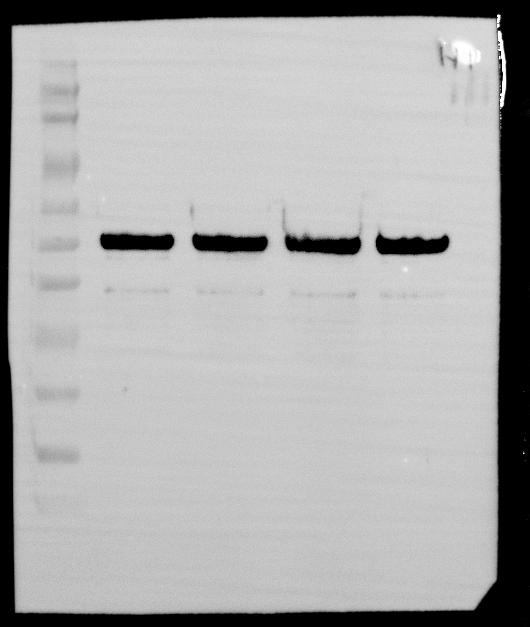


250kD

150kD

70kD

50kD

40kD

35kD

100kD

25kD

20kD

15kD

CON

MOD

ZGP

MET

③
